# Therapeutic Strategies

**DOI:** 10.1186/1129-2377-14-S1-O7

**Published:** 2013-02-21

**Authors:** P Prabhakar

**Affiliations:** 1 Great Ormond Street Hospital, UK

## 

Treatment strategies for childhood headache involves a multi faceted approach. Once a diagnosis is achieved. management choice should include -treatment strategy for acute attacks and prevention. lifestyle management - diet, exercise and sleep, psycho social factors, trigger identification and avoidance, pharmacological agents and neutraceuticals, biofeedback and alternative therapies are discussed.

